# The Effectiveness of a Simulation-Based Menopause Education Program

**DOI:** 10.15766/mep_2374-8265.11625

**Published:** 2026-08-07

**Authors:** Madison Collazo, Aaliyah Meade, Emily Marko, Amanda Slater, Inchi Hu, Carolyn Davis

**Affiliations:** 1 Resident, Department of Obstetrics and Gynecology, Inova Fairfax Medical Campus; 2 Medical Student, University of Virginia School of Medicine, Inova Fairfax Medical Campus; 3 Director, Inova Center for Advanced Medical Simulation, Inova Fairfax Medical Campus; 4 Academic and Research Coordinator, Department of Obstetrics and Gynecology, University of Virginia School of Medicine, Inova Fairfax Medical Campus; 5 Professor, Department of Statistics, College of Engineering and Computing, George Mason University; 6 Assistant Clerkship Director, Department of Obstetrics and Gynecology, University of Virginia School of Medicine, Inova Fairfax Medical Campus

**Keywords:** Menopause, Simulation, Standardized Patient, Obstetrics and Gynecology, Family Medicine, Internal Medicine, OB/GYN, Women's Health

## Abstract

**Introduction:**

Menopause education is often neglected in resident curriculum. A 2017 survey of OB/GYN, family medicine, and internal medicine residents revealed that over 90% of participants considered training in menopause management important, but only 6.8% felt prepared to provide it. We aimed to fill this gap using standardized patient encounters.

**Methods:**

This 2- to 3-hour educational experience was offered at the Inova Center for Advanced Medical Simulation. OB/GYN, family medicine, and internal medicine residents of all PGY levels were eligible. An experienced obstetrician-gynecologist led a didactic session addressing menopausal concerns and management followed by resident engagement with 3 standardized patient encounters. Pre- and postdifferences in knowledge and confidence were compared, as well as performance scores. Statistical methods included a comparison of means using a paired *t* test at a 5% significance level and a 95% CI. Analysis of variance was used to compare differences among specialties.

**Results:**

Participants included 13 OB/GYN, 13 family medicine, and 8 internal medicine residents. Mean differences in pre- and postknowledge tests revealed a mean improvement of 1.5 out of 6 points (95% CI, 1.07–1.93; *P* < .0001) with no significant differences in improvement among specialties. Confidence scores significantly improved across all groups.

**Discussion:**

Following this didactic and simulation session, residents’ knowledge and confidence regarding menopause management improved. Simulation is a valuable tool for supplementing resident learning, particularly for topics not often encountered in resident clinics. This educational experience highlights the utility of interdisciplinary teaching in resident education.

## Educational Objectives

By the end of this activity, learners will be able to:

1. Identify the most common symptoms of menopause.
2. Demonstrate knowledge of nonhormonal treatment of menopausal symptoms through the use of a performance checklist during a standardized patient encounter.
3. Apply evidence-based national guidelines to counsel a standardized patient on hormone replacement therapy, including bioidentical hormones, as measured through a performance checklist.
4. Formulate an appropriate treatment plan for low bone density during a simulated patient encounter.
5. Through role-playing, use shared decision-making to formulate management plans regarding nonhormonal treatment of menopause symptoms, hormone replacement, and abnormal bone density studies (dual-energy x-ray absorptiometry [DEXA]).

## Introduction

There are roughly 20 million women in the US between the ages of 45 and 54 years, representing the bulk of women entering the menopausal transition.^1^ As the US population continues to grow, the number of perimenopausal women presenting to physicians will continue to increase. Many of these patients will continue to rely on their obstetrician-gynecologists for perimenopausal care, whereas some will turn to their family medicine physicians or internists. A 2013 survey of OB/GYN residents showed that only 20.8% reported formal menopause education as part of their residency curriculum. Most participants reported limited knowledge in key menopause topics, including physiology, hormone therapy, nonhormone therapy, bone health, cardiovascular disease, and metabolic syndrome.^2^

A 2017 survey of OB/GYN, family medicine, and internal medicine residents revealed that over 90% of participants felt it was important to be trained in managing menopause, but only 6.8% felt adequately prepared to do so.^3^ A survey of OB/GYN program directors in 2023 revealed that only 31.3% of programs had a dedicated menopause curriculum. Of all programs surveyed, only 23.2% had dedicated menopause clinics or clinics with high volumes of menopause patients, highlighting a gap in residents’ contact with these patients.^4^

In their scoping review of menopausal education among health professionals, Keye et al.^5^ show a paucity of menopause education across medical education. One prior study implemented a telemedicine standardized patient encounter to improve medical students’ recognition of menopausal symptoms with good success.^6^ Another study attempted to fill this gap by utilizing weekly menopause clinics, resulting in improved resident knowledge of menopause topics.^7^

Simulation has been shown to improve medical trainees’ communication skills and comfort discussing sensitive topics. Studies have also shown that case-based learning and standardized patient encounters improve knowledge when compared with traditional teaching methods.^8,9^

To our knowledge, no studies have utilized a series of standardized patient encounters to increase residents’ knowledge and comfort with menopausal symptoms and treatment. While 1 prior study looked at medical students’ ability to interview and examine standardized patients presenting with hot flashes,^6^ we aimed to expand our experience to include abnormal bone density and genitourinary syndrome of menopause, and improve residents’ comfort with counseling patients on management of these topics. Our educational experience aimed to fill a gap in menopause education for residents training in OB/GYN, family medicine, and internal medicine by using standardized patient encounters that address common menopausal concerns.

## Methods

### Development

We developed this simulation as part of an OB/GYN resident research project in conjunction with OB/GYN faculty. We designed the session to include trainees from our institution training in OB/GYN, internal medicine, and family medicine. The standardized patient scenarios were created to address typical menopause concerns, including hot flashes, bone loss, insomnia, weight gain, dyspareunia, and low libido. This project was reviewed and approved by the Institutional Review Board at Inova Fairfax Medical Campus (IRB study U23-02-4968).

### Equipment/Environment

This educational experience took place at the Inova Center for Advanced Medical Simulation at Inova Fairfax Medical Campus. Simulated exam rooms with recording equipment were utilized for the simulated patient encounters. Each room was arranged to allow the standardized patient and provider to sit across from one another in chairs. A discreet camera recorded the encounter, allowing other learners to observe from an outside viewing room. A printed case scenario was available outside of each exam room for review by participants prior to beginning each simulated patient encounter (Appendix A).

### Personnel

An experienced obstetrician-gynecologist with years of clinical practice and teaching experience led a didactic session for all residents prior to the standardized patient encounters. She, along with 2 other OB/GYN faculty members, served as experts during the simulation. These faculty experts completed a performance checklist (Appendix B) for each learner during the simulations to help guide feedback.

Three standardized patients were provided through the simulation center. Standardized patients were chosen based on the case demographic information. The standardized patient cases were developed using the *MedEdPORTAL* Standardized Patient Development Tool.^10^ Each standardized patient was provided with a standardized patient case (Appendix C), which was reviewed with the standardized patient educator. Each was educated about their case using printed materials. An overview of each character, as well as scripted responses to each possible prompt, is available in Appendix C.

One to 2 facilitators were available during each session. These facilitators directed learners to each station and kept time throughout the sessions.

### Implementation

OB/GYN, family medicine, and internal medicine residents of all levels of training were invited to participate in the simulation. Recruitment materials were distributed to each program's academic schedulers (Appendix D). The simulation was offered on 3 dates, once during each program's weekly allotted didactic time. Residents were given the opportunity to opt in or out and were consented as part of the experience. Each session took approximately 2–3 hours to complete. A general workflow and timeline are available in Appendix E.

An experienced obstetrician-gynecologist (Carolyn Davis) led a 1-hour didactic session reviewing common menopausal concerns and management approaches. This presentation was developed using national menopause guidelines and years of clinical experience (Appendix F).

Next, residents were randomly divided into groups of 2–4 learners. Each group rotated through the 3 standardized patient encounters. During each encounter, 1 resident served as the provider while the remaining residents and a faculty expert observed from a viewing room. During each simulation, the faculty expert completed the performance checklist for the learner. Each simulated patient encounter was allowed to run for 10 minutes. As the groups rotated through different encounters, a different resident acted as the provider. The faculty expert remained at their station while the learners rotated.

### Debriefing

Following each completed patient encounter, the remaining members of each small group and faculty expert entered the exam room for a 5-minute feedback session. We used a learner-led, open-ended framework to guide the discussion. A debriefing guide is provided in Appendix G. To start, the learner was asked to assess what went well and what they could have improved on. The standardized patient then provided feedback to the learner from the patient's perspective. Next, the other residents and faculty were given the opportunity to provide feedback to the learner. At this point, the faculty expert reviewed the performance checklist with the group, pointing out areas of strength and opportunities for improvement. After each small group had rotated through all 3 encounters, the entire group of residents reconvened with faculty for 20 minutes to discuss key takeaways.

### Learner Assessment

To assess the effects of the simulation on knowledge and confidence, we started the session with a presession knowledge test and confidence survey. Each resident was assigned a random number. The residents then completed a numbered 6-question knowledge test (Appendix H) to assess baseline knowledge of menopausal care. These questions were developed by a fourth-year medical student using available guidelines with the educational objectives in mind. The questions were reviewed and vetted by the OB/GYN attendings serving as faculty experts. Each resident also completed a numbered 5-question confidence survey (Appendix I) to assess their baseline comfort in managing menopause. The confidence survey used a 5-point Likert scale to determine confidence. The survey was created by the research team with the learning objectives in mind.

During the standardized patient encounters, residents were evaluated by expert faculty using a standardized checklist (see Appendix B) to gauge their comfort discussing menopausal symptoms and counseling patients, as well as their ability to develop appropriate management plans for each concern. The performance checklist was developed by members of the research team—in particular, the director of the simulation center and assistant OB/GYN clerkship director, who regularly develop simulation experiences for medical students and residents at our institution. These evaluations were used to assign a performance score to each participant. At the completion of the session, each participant again completed the knowledge test and confidence surveys.

Statistical methods included a comparison of means using the paired *t* test and chi-squared analysis with a *P* value < .05 considered significant. Mean results from the pre-and postsession knowledge tests were compared. For the confidence survey, the 5-point Likert scale results were compared for participant responses before and after the skills curriculum using binomial data points. Analysis of variance was used to compare differences among specialties. The performance scores were compared by standardized patient case. Finally, any open-ended responses from participants were analyzed qualitatively for themes.

## Results

Thirty-four residents participated in the experience, including 13 OB/GYN residents, 13 family medicine residents, and 8 internal medicine residents. The family medicine participants included PGY-2 and PGY-3 residents. OB/GYN and internal medicine included residents from PGY-1 to PGY-3 levels. The specific demographics for each participant were not recorded.

All 34 residents completed the pre- and postsession knowledge tests; however, 1 participant omitted 3 questions on the presession test. These questions were counted as incorrect. The average presession knowledge score for all participants was 44.11%. The average postsession knowledge score was 69.12%. Overall, knowledge increased by 1.5 points out of 6 (95% CI, 1.0–1.93; *P* < .0001), representing a 25% increase.

The average pre- and postsession knowledge scores were calculated for each group of residents. All 3 groups of residents had an improvement in knowledge (Figure). A 1-way ANOVA showed no significant difference in knowledge improvement among specialties.

**Figure.**
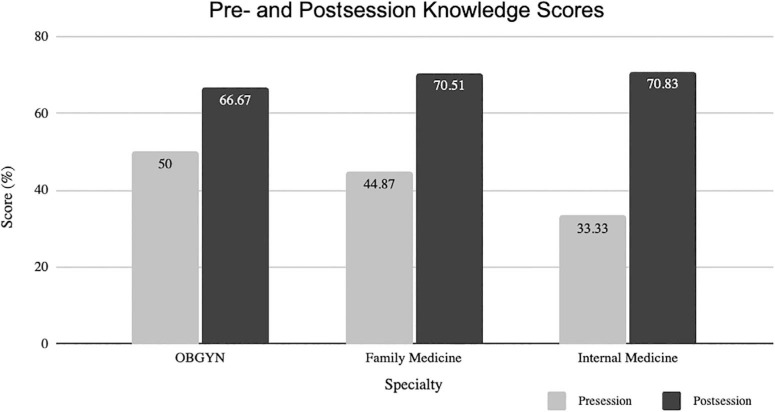
Pre- and postsession knowledge scores by specialty.

Average pre- and postsession confidence scores were also compared. Participants rated their confidence for each prompt on a scale of 1 (very confident) to 5 (very unconfident). Thirty-two participants completed the presession confidence survey, whereas the full 34 participants completed the postsession confidence survey. The missing results were omitted from the calculated average.

There was a statistically significant increase in confidence across all questions (Table 1). One-way ANOVA revealed no significant differences between improvement in pre- and postsession confidence scores when comparing all specialties.

**Table 1.**
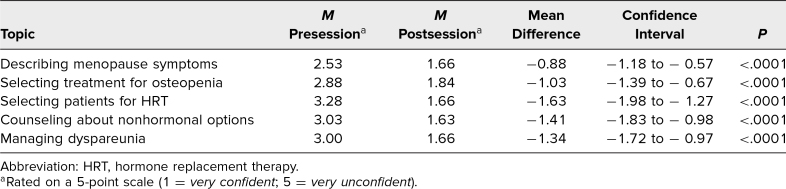
Mean Difference in Confidence Scores Among All Participants (*N* = 34)

Performance scores determined by checklist during the standardized patient encounters are summarized in Table 2. In the family medicine group, several residents were able to act as the provider in more than 1 standardized patient case. In other groups, such as OB/GYN, some residents never played the provider role due to time constraints. Six family medicine residents, 2 internal medicine residents, and 3 OB/GYN residents served as the provider in each of the three standardized patient cases. Regardless, all participants observed the encounters. In general, residents participating in the case related to abnormal bone density had lower performance scores. This is attributed to the lower relevance in discussing certain checklist items in a case related to bone density (vaginal bleeding pattern, counseling on bioidentical hormones, etc.). Overall, residents scored very well on checklist items 11 and 12, demonstrating their knowledge of hormonal and nonhormonal treatments for menopausal symptoms and their ability to accurately interpret and counsel patients regarding an abnormal bone density scan. Residents tended to score lower on their ability to counsel patients regarding bioidentical hormones. Through the encounters, the participants used shared decision-making to develop appropriate management plans with the standardized patients.

**Table 2.**
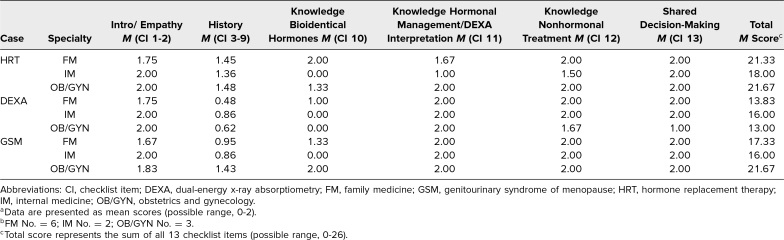
Performance Scores for Each Standardized Patient Case by Learner Specialty^a^

Open-ended responses were collected from residents participating in the sessions. In general, residents felt that the session was a helpful review for this important topic. Several responses are shared next:

• “This was a great session and review and it would be very helpful for the early second-year FM [family medicine] residents before they start their outpatient training. So many of the patients are perimenopausal.”
• “I am happy to have had a chance to practice these conversations through simulation and refresh the knowledge base!”

## Discussion

With an aging population that is now more than ever recognizing the impact of the menopausal transition on a woman's life, physicians who provide primary care must be educated in these topics and prepared to manage their patients’ needs. Traditional resident curriculum across multiple specialties has failed to provide residents with the necessary knowledge and experience to properly care for these patients. Although there have been recent efforts to increase awareness and education surrounding menopausal needs, residents training in primary care specialties and OB/GYN continue to lack the adequate knowledge and tools to appropriately care for menopausal patients.

We leveraged simulation to address a gap in resident education. Our innovative multimodal menopause education session provided residents with valuable practice in recognizing common menopausal concerns, interpreting test results, discussing sensitive topics with patients, and counseling patients on the risks and benefits of various treatment options. Trainees demonstrated understanding and application of national guidelines by appropriately counseling patients on hormonal and nonhormonal treatment options and using shared decision-making to formulate management plans for menopausal concerns. Following this didactic and simulation session, residents training in OB/GYN, family medicine, and internal medicine showed improvements in both knowledge and confidence.

This simulation was unique in that it involved residents from multiple specialties, highlighting the benefits of multidisciplinary learning. Our simulation would have been further strengthened by grouping residents from different specialties together to allow residents to learn from one another; however, this was not possible due to each groups’ limited availability away from clinical duties. As with menopause, many medical topics span multiple disciplines, presenting opportunities for further interdisciplinary education and simulation.

While this curriculum showed promising results, our data also has limitations. Although our results showed consistent improvement in knowledge and confidence, these improvements only reflect short-term changes. Our data would be strengthened by testing knowledge retention at 6 weeks and 6 months through repeating the knowledge test and standardized patient encounters. In addition, it would be helpful to survey participants several months after the simulation to assess its lasting impact.

There is also an element of selection bias, as participation was optional. Positive results may reflect participation by residents interested in learning about this topic. A future study involving all residents would help reduce this bias.

Our results may also have been affected by differences in training levels among the residents. While all trainee levels participated, we did not track the demographics of the different resident groups. Therefore, an uneven split of training levels may have affected the results of the different groups. We do know that only PGY-2 and PGY-3 family medicine residents participated, whereas the other specialties included interns. It is possible that excluding interns from the family medicine data may have contributed to higher mean scores. Additionally, while family medicine and internal medicine had representatives from the PGY-3 class, or final year of training, there were no PGY-4 participants from OB/GYN. In theory, PGY-4 OB/GYN residents preparing to graduate are likely to have greater baseline knowledge of menopause due to independent study in anticipation of individual practice after residency. This exclusion may have contributed to lower mean scores from the OB/GYN group. We may have gathered meaningful data regarding the impact of this experience by comparing results of the knowledge test, confidence survey, and performance checklist between residents of the same specialty and training year who completed the simulation versus those who did not.

While completing 1 menopause simulation session is valuable, repeated training is likely more beneficial. Our OB/GYN residency program has implemented this session into the residents’ didactics curriculum, allowing residents to practice these skills yearly. Our hope is that other institutions will adopt this curriculum to improve their residents’ knowledge and confidence in treating menopausal needs.

## Appendices


Door Notes.docxPerformance Checklist.docxStandardized Patient Cases.docxRecruitment Email.docxWorkflow and Timeline.docxMenopause Presentation.pptxFaculty Debriefing Guide.docxKnowledge Test with Answers.docxConfidence Survey.docx
*All appendices are peer reviewed as integral parts of the Original Publication.*
