# Supplementary material for: The Effectiveness of a Simulation-Based Menopause Education Program
Source: MedEdPORTAL. 2026 Aug 7;22:11625. doi: 10.15766/mep_2374-8265.11625 (PMC13447422; doi:10.15766/mep_2374-8265.11625)
Supplement: Supplementary file 1 [file mep_2374-8265.11625-s001.zip › D. Recruitment Email.docx]

As an OB/GYN, Family Medicine, or Internal Medicine resident, we would like to invite you to participate in a simulation-based program in educating residents about menopause.

Most OB/GYN, Family Medicine, and Internal Medicine physicians will take care of peri- and post-menopausal patients after residency. Unfortunately, menopausal care is typically not a formalized part of resident education. As a result, physicians often must rely on self-directed learning after residency to prepare to appropriately treat these patients.

The purpose of the simulation is to increase resident knowledge, confidence, and competency in caring for menopausal patients. Residents from the OB/GYN, Family Medicine, and Internal Medicine programs will first take an initial confidence survey and a pre-test about baseline knowledge of menopausal symptoms and management of common concerns. They will then participate in a lecture about menopause and current guidelines in management. Next, the residents will participate in a simulation-based session where they will apply what they have learned to counsel and treat standardized patients. Following the session, residents will repeat the confidence survey and post-test to demonstrate what they have learned.
