# Supplementary material for: The Effectiveness of a Simulation-Based Menopause Education Program
Source: MedEdPORTAL. 2026 Aug 7;22:11625. doi: 10.15766/mep_2374-8265.11625 (PMC13447422; doi:10.15766/mep_2374-8265.11625)
Supplement: Supplementary file 1 [file mep_2374-8265.11625-s001.zip › E. Workflow and Timeline.docx]

Menopause Education Experience Workflow and Timeline
