# Supplementary material for: The Effectiveness of a Simulation-Based Menopause Education Program
Source: MedEdPORTAL. 2026 Aug 7;22:11625. doi: 10.15766/mep_2374-8265.11625 (PMC13447422; doi:10.15766/mep_2374-8265.11625)
Supplement: Supplementary file 1 [file mep_2374-8265.11625-s001.zip › G. Faculty Debriefing Guide.docx]

**Appendix F. Faculty Debriefing Guide**

**CREATE A SAFE ENVIRONMENT**

1. Set the tone to hold the debrief using non-judgmental language, ensuring an environment that allows learners to feel comfortable discussing their performance and sharing their experience.
2. Set expectations for what will be discussed in the debrief.
3. Foster student/resident-led discussion: use open-ended and reflective questions to guide the conversation.

**COMMUNICATION**

Have learners share about their SP encounters:

1. What went well and why?
2. What did you find challenging and how would you differ your approach?
3. Were you able to support your recommendations for treatment to the SP?
4. How did you approach SP hesitations about treatment?

**REFLECTIVE PRACTICE**

1. What were the key takeaways?
2. Was there anything that made you uncomfortable in counseling the patient about hormones for menopause symptoms or medications to prevent bone loss?
3. Are there ways to be efficient in counseling a patient during an office visit?
4. Were you surprised to learn anything during this exercise?

**SUMMARIZATION**

1. Review common themes from the simulation exercise.
2. Recommend or provide supplemental education tools to learners if necessary to fortify	knowledge.

Sources:

1. Debriefing the Interprofessional Team in Medical Simulation. Irim Salik; John T. Paige, Treasure Island (FL): [StatPearls Publishing](https://www.statpearls.com/); 2025 Jan
2. <https://cisl.stanford.edu/design-a-program/create-an-effective-curriculum/art-of-debriefing.html>
