# Supplementary material for: The Effectiveness of a Simulation-Based Menopause Education Program
Source: MedEdPORTAL. 2026 Aug 7;22:11625. doi: 10.15766/mep_2374-8265.11625 (PMC13447422; doi:10.15766/mep_2374-8265.11625)
Supplement: Supplementary file 1 [file mep_2374-8265.11625-s001.zip › H. Knowledge Test with Answers.docx]

**H. Knowledge Test (Pre and Post):**

1. A 53-year-old nulligravid woman reports to her gynecologist’s office for evaluation of

“sweating episodes”. The episodes began six months ago and have interfered with her

ability to focus while at work. These episodes recently began occurring at night. Her last menstrual period was 14 months ago. Medical history includes asthma and depression.

Medications include albuterol and fluoxetine. She endorses heat intolerance, hot flashes,

fatigue, and occasional headaches all starting six months ago. She is feeling more anxious. She denies any fever, chills, weight loss, unexplained vaginal bleeding, palpitations, chest pain, cold intolerance or skin changes. Her vitals are within normal limits during this visit. The patient’s symptoms are most likely associated with which of the following diagnoses?

a) Medication side effect

b) Hyperthyroidism

c) Pheochromocytoma

d) Nodular sclerosing Hodgkin lymphoma

e) Vasomotor symptoms of menopause

f) Panic disorder

2. A 66-year-old, postmenopausal woman reports for a follow-up appointment with her

healthcare provider to discuss the results of her most recent bone density studies. She

expresses concern about her risk of falling and fracturing her hip like her mother. She

denies any personal history of prior fractures. Her dual-energy x-ray absorptiometry

(DEXA) results show a femoral neck T-score of -2.0. Weight is 135 lbs (61.2 kg), height

is 154.9 cm (5 ft. 1 in.) and BMI is 25.5 kg/m^2^. She has no medical problems and takes

no medications. She is a lifetime non-smoker and occasionally drinks alcohol. During the

visit, a FRAX score was calculated and showed her 10-year probability of a hip fracture to be 2.1% and major osteoporotic fracture to be 20%. Given this information, what is the next best step in management for this patient?

a) Recommend initiation of bisphosphonate therapy

b) Recommend yearly or biannual DXA scans to monitor bone mineral density

c) Recommend initiation of Vitamin D and calcium supplements

d) Recommend a home evaluation to determine risk for falls at home

3. A 60-year-old postmenopausal woman returns to the office with a new concern for chest

discomfort after starting oral bisphosphonate therapy for osteoporosis of the spine.

She describes the discomfort as a burning sensation in the center of her chest. It is

worsened by lying flat and bending forward. Review of systems is positive for increased

belching. She reports taking the medication exactly as instructed by the pharmacist. Her

vital signs are normal. Physical examination unremarkable. EKG showed sinus rhythm

with no abnormalities. Medical history is non-contributory. Family history positive for

mother with breast cancer at age 70. What is the next best step in management?

a) Prescribe a trial proton pump inhibitor

b) Ask patient to describe how they take their oral medication

c) Switch to a selective estrogen receptor modulator for therapy

d) Continue with the same medication regimen

e) Switch to once a month ibandronate for therapy.

4. A 54-year-old woman reports to her gynecologist to discuss treatment options for

decreased libido, dyspareunia, sleep disturbances and changes in mood. Her last menstrual period occurred 13 months ago. During the visit, she pulls out an advertisement for a newly available, personalized compounded bioidentical hormone treatment for postmenopausal women. She asks the gynecologist their opinion on this new type of medication for the treatment of common menopausal symptoms. Which of the following responses below reflects the most current, evidence-based guideline recommendations on the efficacy of compounded bioidentical menopausal hormone therapy (cMHT) in comparison to conventional FDA-approved MHT?

a) Conventional MHT is the preferred recommendation over cMHT for the treatment of common menopausal symptoms given the available efficacy and safety data.

b) There are FDA-approved options for bioidentical conventional MHT.

c) Current evidence is lacking to support claims of superior efficacy of cMHT over FDA-

approved MHT.

d) cMHT preparations (creams, tablets, pellet implants, injections) lack safety data

and have variable bioavailability and bioactivity properties which expose patients to risks of possible under and over-dosage of these compounds.

e) There is lacking evidence to support the increased efficacy or safety associated with the use of salivary, serum or urinary testing and monitoring of patients taking cMHT regimens.

f) All of the above

5. A 58-year-old nulligravid woman presents to the clinic with concern for burning with

urination and urinary frequency. Her last menstrual period was 4 years ago. For the past four years, she has experienced vulvovaginal dryness and mild pruritus. Medical history is positive for three urinary tract infections in the last 6 months. She has no surgical history. Her only medication is a daily women’s multivitamin, and she is not sexually active. Vital signs are normal. Physical examination is notable for reduced vulvar skin elasticity, pale and dry vaginal mucosa with minimal rugae and erythematous patches. There is no leakage of urine on Valsalva. Urinalysis is positive for trace leukocyte esterase and trace protein. In addition to evaluating further with a urine culture, what additional diagnosis and treatment recommendation should be shared with this patient?

a) Genitourinary syndrome of menopause, topical non-hormonal moisturizers and lubricants

b) Genitourinary syndrome of menopause, topical estrogen cream

c) Vulvovaginal candidiasis, topical azole therapy

d) No additional diagnosis or treatment recommendation necessary

6. A 52-year-old patient is experiencing extreme hot flashes that occur frequently at night and often prevent restful sleep. Her last period was 8 months ago and she has been told that she is menopausal by lab work. Her past medical history is positive for Stage 1 breast cancer that was diagnosed at age 50. She is desperate for options to help with the hot flashes but was told by her oncologist that she could not take hormones. She has also noticed a drop in libido. Which of the following would be the best choice of treatment for this patient?

a) Black Cohosh

b) Aerobic exercise several times a week

c) Gabapentin

d) Phytoestrogens (isoflavones in soybeans)

e) Venlafaxine

**Answers and Explanations:**

1. **Correct Answer: E. Vasomotor symptoms of menopause**

Explanation: The patient’s primary concern and associated positives on review of systems are most consistent with vasomotor symptoms (VMS) of menopause. These symptoms include sweating, heat flashes/hot flashes, heat intolerance and night sweats. Recent data suggests nearly three out of every four postmenopausal individuals report experiencing vasomotor symptoms; and at least one of every four of these patients younger than 55 years of age report their VMS severity as ranging from ‘moderately to severely bothersome’.^1^ Vasomotor symptoms of menopause are a recognized clinical indication for menopausal hormone therapy (MHT).

E.O.1: Identify the most common symptoms of menopause.

2. **Correct Answer: A. Recommend initiation of bisphosphonate therapy.**

Explanation: The patient’s DEXA results indicate osteopenia of the femoral neck (T

score between -1 and -2.5) as opposed to osteoporosis (T score ≤-2.5). Osteopenia

alone is not an indication for initiation of pharmacotherapy as stated in multiple

management guidelines; however, the patient presented with multiple fracture risk

factors including: advanced age (>65 yo), postmenopausal and parental history of

fracture. Her risk factors along with her calculated 10-year probability of a major

osteoporotic fracture being ≥20% should prompt immediate consideration of either

bisphosphonates or non-bisphosphonate therapy to reduce risk of fracture.

Other osteoporosis risk factors include low body weight (<58kg), estrogen deficiency (i.e.

premature menopause, oophorectomy), history of inflammatory disorders (i.e.

rheumatoid arthritis), Caucasian or Asian descent, malabsorption disorders, chronic liver

or kidney disease, hypercortisolism, hyperthyroidism, hyperparathyroidism, Vit D

deficiency, inadequate calcium intake, current/prior hip or vertebral fracture, smoking,

excessive alcohol intake, glucocorticoid usage, anticonvulsants and other medications.

Primary osteoporosis (T score ≤-2.5) is an indication for immediate consideration of

either bisphosphonates or non-bisphosphonate therapy to reduce risk of fracture. Home

evaluation to determine risk of falls at home would be done for someone who has limited

mobility, balance issues, or a cognitive disorder.

E.O.3: Demonstrate knowledge of appropriate treatment for low bone density during a simulated patient encounter and knowledge test.

3. **Correct answer: C. Switch to a selective estrogen receptor modulator for therapy**

Explanation: Esophagitis is a complication associated with oral bisphosphonates

(alendronate, risedronate, etidronate, tiludronate, and ibandronate) therapy. Patients

should be counseled on the appropriate methods for taking oral bisphosphonates to

decrease the chances of experiencing this complication (swallow medication with

adequate amounts of water and remain upright for 30 minutes or more). For

postmenopausal patients without current or prior venous thromboembolism disorders

(i.e. pulmonary embolism, DVT, retinal vein thrombosis) who cannot tolerate oral

bisphosphonate therapy, selective estrogen receptor modulator (SERM) therapy with

raloxifene can be considered. Raloxifene is a SERM that acts as an estrogen agonist on

bone to decrease postmenopausal bone remodeling. Raloxifene has been found to decrease the risk of breast cancer and is approved for prevention and treatment of vertebral fractures but not non-vertebral fractures.

E.O.3: Demonstrate knowledge of appropriate treatment for low bone density during a simulated patient encounter and knowledge test.

4. **Correct answer: F. All of the above**

Explanation: The ACOG Committee Opinion No. 532 outlines the major issues of

concern surrounding the use of compounded bioidentical menopausal hormone therapy

in treatment of menopausal symptoms for postmenopausal patients. The committee

opinion also discusses how to navigate patient counseling when comparing conventional

FDA approved MHT to compounded bioidentical menopausal hormone therapy options.

As the answer choices highlight, current evidence is lacking to support claims of superior

efficacy or safety profiles of compound bioidentical hormone use.^3^ Clinicians should

inform patients that MHT is proven to be safe and effective by the FDA and provide

evidence-based recommendations while helping patients select the best treatment given

their individual patient factors. There are FDA approved options for MHT that include bioidentical estradiol and natural progesterone.

E.O. 2: Demonstrate competency using a checklist regarding evidence-based national

guidelines for hormonal and nonhormonal treatment of menopause symptoms.

5. **Correct answer: B. Genitourinary syndrome of menopause, topical estrogen cream.**

Explanation: The patient is presenting with common symptoms and signs of

genitourinary syndrome of menopause (GSM), previously referred to as atrophic

vaginitis. GSM is caused by estrogen deficiency. Estrogen deficiency (i.e. in cases of

menopause or surgical removal of ovaries) leads to decreased collagen production in

the estrogen sensitive tissues of the urethra and bladder trigone which can induce

urgency incontinence. Estrogen deficiency also leads to decreased vaginal glycogen

content which results in loss of normal vaginal microflora and an elevated vaginal pH

increasing patients’ risk of urinary tract infections. With decreased glycogen content, the

vulvar skin is at higher risk of breaking or fissuring which can create discomfort when

exposed to acidic urine. First-line treatment for GSM includes topical non-hormonal

vulvovaginal moisturizers and lubricants. For patients with severe GSM symptoms or

those who fail to respond to first-line therapies, low dose-vaginal estrogen options may

be considered.^4^

E.O.1: Identify the most common symptoms of menopause.

E.O. 2: Demonstrate competency using a checklist regarding evidence-based national

guidelines for hormonal and nonhormonal treatment of menopause symptoms.

6. **Correct Answer: C. Gabapentin**

Explanation: Given the lack of current evidence of effectiveness and the possible

hepatotoxicity concerns, health care professionals should not recommend black cohosh

for the treatment of hot flashes. A comparative effectiveness review on menopausal

symptoms supported by the Agency for Healthcare Research and Quality found that

there was low strength of evidence that isoflavones improve vasomotor symptoms

compared with placebo. SSRIs and SNRIs have been found to be effective for hot

flashes but there are side effects, including decreased libido. Gabapentin has been found effective for hot flash reduction. Due to possible somnolence caused by gabapentin, it can be helpful for patients with night time hot flashes that are interrupting their sleep. There is insignificant evidence that exercise causes a decrease in hot flashes.^5^

E.O. 2: Demonstrate competency using a checklist regarding evidence-based national

guidelines for hormonal and nonhormonal treatment of menopause symptoms.

***Note: For the purposes of our educational experience, Appendix H represents the actual multiple-choice questions provided to participants. For replication, we would recommend making question #5 a two-part question for improved clarity. As an example:**

A 58-year-old nulligravid woman presents to the clinic with concern for burning with

urination and urinary frequency. Her last menstrual period was 4 years ago. For the past four years, she has experienced vulvovaginal dryness and mild pruritus. Medical history is positive for three urinary tract infections in the last 6 months. She has no surgical history. Her only medication is a daily women’s multivitamin, and she is not sexually active. Vital signs are normal. Physical examination is notable for reduced vulvar skin elasticity, pale and dry vaginal mucosa with minimal rugae and erythematous patches. There is no leakage of urine on Valsalva. Urinalysis is positive for trace leukocyte esterase and trace protein.

1. What is the most likely diagnosis?
   a. Genitourinary syndrome of menopause
   b. Vulvovaginal candidiasis
   c. Lichen planus
   d. No additional diagnosis
2. What treatment would you recommend for this patient?
   a. Topical non-hormonal moisturizers and lubricants
   b. Topical estrogen cream
   c. Topical azole therapy
   d. Topical steroid cream
   e. No treatment recommendation necessary

**References:**

1. Davis, S.R., Baber, R.J. Treating menopause — MHT and beyond. Nat Rev Endocrinol 18, 490–502 (2022). <https://doi.org/10.1038/s41574-022-00685-4>

2. US Preventive Services Task Force; Curry SJ, Krist AH, Owens DK, Barry MJ, Caughey AB, Davidson KW, Doubeni CA, Epling JW Jr, Kemper AR, Kubik M, Landefeld CS, Mangione CM, Phipps MG, Pignone M, Silverstein M, Simon MA, Tseng CW, Wong JB. Screening for Osteoporosis to Prevent Fractures: US Preventive Services Task Force Recommendation Statement. JAMA. 2018 Jun 26;319(24):2521-2531. doi: 10.1001/jama.2018.7498. PMID: 29946735.

3. Compounded bioidentical menopausal hormone therapy. Committee Opinion No. 532. American College of Obstetricians and Gynecologists. Obstet Gynecol 2012;120:411–5.

4. Palacios S, Mejía A, Neyro JL. Treatment of the genitourinary syndrome of menopause. Climacteric. 2015;18 Suppl 1:23-9. doi: 10.3109/13697137.2015.1079100. PMID: 26366797.

5. McGarry, K., Geary, M., & Gopinath, V. (2018). Beyond estrogen: Treatment options for hot flashes. *Clinical Therapeutics*, *40*(10),1778–1786.https://doi.org/10.1016/j.clinthera.2018.08.010
