# Supplementary material for: The Effectiveness of a Simulation-Based Menopause Education Program
Source: MedEdPORTAL. 2026 Aug 7;22:11625. doi: 10.15766/mep_2374-8265.11625 (PMC13447422; doi:10.15766/mep_2374-8265.11625)
Supplement: Supplementary file 1 [file mep_2374-8265.11625-s001.zip › I. Confidence Survey.docx]

**I. Confidence Survey (Pre and Post):**

Please rate your confidence level in providing menopausal care patient counseling for each of the following questions:

**1. How confident do you feel describing the full scope of menopause symptoms?**

□ Very confident □ Somewhat confident □ Neutral □ Somewhat unconfident □ Very unconfident

**2. How confident do you feel in your ability to appropriately select treatment for a patient presenting with osteopenia?**

□ Very confident □ Somewhat confident □ Neutral □ Somewhat unconfident □ Very unconfident

**3. How confident do you feel in selecting appropriate patients to receive hormone replacement therapy?**

□ Very confident □ Somewhat confident □ Neutral □ Somewhat unconfident □ Very unconfident

**4. How confident do you feel in counseling patients about non-hormonal options for managing menopausal symptoms?**

□ Very confident □ Somewhat confident □ Neutral □ Somewhat unconfident □ Very unconfident

**5. How confident do you feel managing dyspareunia in a menopausal patient?**

□ Very confident □ Somewhat confident □ Neutral □ Somewhat unconfident □ Very unconfident
