# Supplementary material for: The Effectiveness of a Simulation-Based Menopause Education Program
Source: MedEdPORTAL. 2026 Aug 7;22:11625. doi: 10.15766/mep_2374-8265.11625 (PMC13447422; doi:10.15766/mep_2374-8265.11625)
Supplement: Supplementary file 1 [file mep_2374-8265.11625-s001.zip › A. Door Notes.docx]

**Appendix A: Door Notes**

**Case 1:**

**Olivia Johnson is a patient who scheduled an appointment to discuss some symptoms she has been having.**

**Clinical Situation:**

She is a 51 y/o female patient with spacing cycles, hot flashes, difficulty sleeping, and weight gain.

**Associated symptoms***:*

She will get hot without warning and break out in a sweat. Sometimes during these episodes, she will feel palpitations. She has been noticing more fatigue and irritability.

Past Medical History: Hypothyroidism

Medications: Synthroid 50 mcg qd

**Physical exam:**

Stable vital signs - HR 80, BP 122/78, T 98.6, RR 20, Pulse ox 98%

All else is normal.

**Resident Tasks:**

1. Perform a gynecology interview (no physical exam).
2. Demonstrate knowledge of evidence-based national guidelines for hormonal and nonhormonal treatment of menopause symptoms through counseling.
3. Explain the risks, benefits and complications of, and the alternatives to use of hormone replacement.
4. Demonstrate shared decision-making about treatment of menopausal symptoms.

**You have up to 10 minutes with the patient.**

**Case 2:**

**Felicia Watson is a 60 y/o patient who scheduled an appointment to discuss the results of her recent DEXA scan.**

**Clinical Situation:**

She is a 60 y/o female patient who has not had a period in 8 years and is “done” with menopause. She went to a free bone density screening clinic and she was told she had osteopenia and to follow-up with her doctor. The report is attached.

Past Medical History: Mild hypertension, GERD

Fam Med Hx: Mother had breast cancer age 64

Father hip fracture from a fall at age 78

Medications: HCTZ 25 mg qd

**Physical exam:**

Stable vital signs - HR 80, BP 135/85, BMI 19

**Resident Tasks:**

1. Perform a gynecological interview (no physical exam).
2. Interpret a bone density study (DEXA).
3. Discuss medical treatment of low bone density with a patient presenting with an abnormal DEXA scan.
4. Demonstrate shared decision-making about management of the abnormal bone density study (DEXA).

**You have up to 10 minutes with the patient.**

**Bone Density Report**

**Indications: Postmenopausal, History of parent with fractured hip**

**Location:**	**T-score**	**Z-score**	**Classification**

**PA Spine**	**-2.4**	**-1.3**	**Low Bone Density**

**Fem Neck**	**-1.8**	**-0.7**	**Low Bone Density**

**Total Hip**	**-1.1**	**-0.4**	**Low Bone Density**

**FRAX	WHO Fracture Risk Assessment Tool**

**The 10-year probability of fracture**

**Major Osteoporotic Fracture**	**21%**

**Hip Fracture**	**1.9**
**Case 3:**

**Doretha Patel is a known patient of yours who scheduled an appointment to discuss some problems she is having with her spouse. You have done an exam and now will speak with her.**

**Clinical Situation:**

She is a 55 y/o female patient whose last menstrual period was at age 52. Over the last year she has noticed more pain with intercourse.

**Associated symptoms***:*

She has noticed more vaginal dryness and sex is painful, even when she uses KY jelly. She often just tells her partner that she has a headache and can’t have sex because she is just not that interested.

Past Medical History: mild asthma

Past Surgical History: Laparoscopic hysterectomy at age 42 - fibroids

Medications: Singulair 10 mg qd

**Physical exam:**

Vital signs - HR 78, BP 122/75, T 98.6, RR 20

The exam you just performed reveals:

External genitalia – normal female anatomy

Vagina - pale and dry vulvovaginal mucosa with petechiae, loss of vaginal rugae, no discharge seen, absent cervix.

Bimanual exam – absent uterus, no adnexal masses.

**Resident Tasks:**

1. Perform a gynecology interview.
2. Demonstrate knowledge of evidence-based national guidelines for hormonal and nonhormonal treatment of menopause symptoms through counseling.
3. Explain the risks, benefits and complications of, and the alternatives to use of hormone replacement.
4. Demonstrate shared decision-making about treatment of menopausal symptoms.

**You have up to 10 minutes with the patient.**
