# Supplementary material for: The Effectiveness of a Simulation-Based Menopause Education Program
Source: MedEdPORTAL. 2026 Aug 7;22:11625. doi: 10.15766/mep_2374-8265.11625 (PMC13447422; doi:10.15766/mep_2374-8265.11625)
Supplement: Supplementary file 1 [file mep_2374-8265.11625-s001.zip › B. Performance Checklist.docx]

| **Case: Menopause Counseling** | **Score of 2** | **Score of 1** | **Score of 0** |
| --- | --- | --- | --- |
| 1. Introduces self, explains his/her role | Introduces self to patient and explains his/her role. | Introduces self to patient but does not explain his/her role. | Does not introduce self to patient |
| 2. Demonstrates active listening and empathy towards patient.   - Direct eye contact - Allow her to describe her symptoms - Give reassuring verbal cues - Reassure the patient that her symptoms are common and not unusual for menopause | Demonstrates active listening and empathy by including 3 of the items | Demonstrates active listening and empathy by including 1 or 2 of the items | Does not demonstrate active listening or empathy. |
| 3. Inquires about hot flashes and impact on lifestyle   - Frequency of hot flashes - Impact on daily schedule - Has she done anything to alleviate them? | Inquires about hot flashes and includes 3 of the items | Inquires about hot flashes and includes 1 - 2 of the items | Does not inquire about hot flashes. |
| 4. Inquires about vaginal bleeding pattern (frequency and amount)   - When was her LMP - Has there been irregular bleeding - Frequency of bleeding - Amount of bleeding - Inquires if sexually active | Inquires about vaginal bleeding by including 2 or more items | Inquires about vaginal bleeding and includes 1 of the items | Does not inquire about vaginal bleeding. |
| 5. Inquires about vaginal dryness and impact on lifestyle   - Impact on lifestyle - Has she tried OTC products? - Has she tried hormonal products? | Inquires about vaginal dryness and includes 2 or more of the items | Inquires about vaginal dryness but does not ask about impact on lifestyle or products that she has tried. | Does not inquire about vaginal dryness. |
| 6. Inquires about sleeping pattern and impact on lifestyle | Inquires about sleeping pattern and impact on lifestyle. | Inquires about sleeping pattern but not the impact it has on lifestyle. | Does not inquire about sleeping pattern. |
| 7. Inquires about/reviews past medical history   - Hx of blood clots - Hx prior fractures - Allergies - Hx of stroke - Hx of heart disease - Hx GERD/ulcer/GI disorder - Hx kidney disease - Hx of cancer - Hx thyroid disorder | Inquires about 4 or more items | Inquires about 2 or 3 of the items | Does not inquire or only inquires about 1 of the items |
| 8. Inquires about/reviews family history   - Hx breast cancer - Hx osteoporosis - Hx of hip fracture in either parent - Hx of blood clots - Hx of ovarian cancer | Inquires about 3 or more items | Inquires about 1 or 2 items | Does not inquire about family history. |
| 9. Inquires about breast cancer risk and screening   - Personal hx of breast cancer - Fam hx of breast cancer - Fam hx ovarian cancer - Most recent mammogram | Inquires about 2 or more items | Inquires about 1 item | Does not inquire about breast cancer risk or screening. |
| 10. Answers questions about compounded bio-identical hormones | Explains the possible risks of compounded bio-identical hormones. |  | Does not explain the possible risk of compounded bio-identical hormones |
| 11. Based on scenario, demonstrates knowledge of one of the following:  **Hormonal treatment of menopause symptoms**   - Explains national guidelines for use of hormone replacement - Explains the difference between systemic and locally acting hormones for menopause symptoms   **Interpretation of a bone density test.**   - Explains the significance of the DEXA report and her higher risk of fracture. | Discusses at least 1 of the items related to the appropriate case |  | Does not demonstrate knowledge of the following: 1) hormonal treatment of menopause symptoms  2) Interpretation of a bone density test. |
| 12. Demonstrates knowledge of non-hormonal treatments for one of the following:  **Menopause symptoms**   - Alternative medications for hot flashes (SSRIs, gabapentin) - Explains OTC options for vaginal dryness - Discusses locally acting medications for vaginal and vulvar symptoms - Discusses lifestyle changes to treat symptoms of menopause (exercise, medication, stress reduction, dietary)   **Abnormal bone density**   - Medications to treat bone loss (biphosphonates, denosumab) - Recommends calcium, Vit D, and weight bearing exercise | Discusses with patient 2 or more items | Discusses with patient 1 of the items | Does not demonstrate knowledge. |
| 13. Uses shared decision-making for treatment of one or more of the following:   - Menopausal symptoms - Hormone replacement - Management of the abnormal bone density study (DEXA). | Offers the options available that are evidence based and allows the patient to choose which option she prefers. If she asks for guidance, you may give her your recommendation. |  | No shared decision making occurs. Provider picks an option without explaining the other options or without explaining the risks. |
| Total Score (max 26) |  |  |  |
