# Supplementary material for: The Effectiveness of a Simulation-Based Menopause Education Program
Source: MedEdPORTAL. 2026 Aug 7;22:11625. doi: 10.15766/mep_2374-8265.11625 (PMC13447422; doi:10.15766/mep_2374-8265.11625)
Supplement: Supplementary file 1 [file mep_2374-8265.11625-s001.zip › C. Standardized Patient Cases.docx]

**Appendix C: Standardized Patient Cases**

**Case 1: *MedEdPORTAL* Standardized Patient Case Development Tool**

**Date**: 2/20/2023

**Primary Case Author:** Carolyn Davis, MD

**Secondary Case Author**: Madison Collazo, MD

**Standardized Patient Educator**: Meghan Willson, BS, CHSE

**Name of Case:** SP Script Case 1 – Menopausal Counseling

**Name of Educational and/or Assessment Activity:** *The Effectiveness of a Simulation-Based Menopause Education Program*

**Patient Name:** Olivia Johnson

**Chief Complaint:** Hot flashes and difficulty sleeping

**Most Likely Diagnosis and Differential With Rationale From History and/or Physical Exam**: Menopause Transition

**Challenge Question:** At 5 min. announcement, if you haven’t brought up this question already, you should find a way to ask “What about the “safe natural bioidentical hormones”? My friend tells me that her pharmacist makes hormone pellets and cream for her. (Please say both sentences so that the resident learner knows that you are referring to compounded bioidentical hormones.)

**Domains: Check all that apply**

X	Professionalism

X	Communication and Interpersonal Skills

X	Medical History

- Physical Exam

X	Shared Decision-Making

X	Patient Education

X	Clinical Reasoning

- Documentation
- Handoff
- Presentation
- Other:

**Type and Level of Learner**: OBGYN, Internal Medicine, and Family Medicine residents

**Case Objectives**: Please list specific objectives for each of the domains you have checked above:

1. Identify the most common symptoms of menopause.
2. Demonstrate competency using a performance checklist regarding evidence-based national guidelines for hormonal and nonhormonal treatment of menopause symptoms.
3. Demonstrate knowledge of appropriate treatment for low bone density during a simulated patient encounter and knowledge test.
4. Through role-playing, demonstrate shared decision-making about treatment of menopausal symptoms, hormone replacement, and management of the abnormal bone density study (DEXA).

| **SETTING**: outpatient, in patient, ED, home, nursing home, rehab, group, etc. | Outpatient exam room |
| --- | --- |
| **PATIENT PROFILE**: Information about the “patient” that helps select an SP and helps the learner get an understanding of them as a person. SP will know more information about the patient than learner will ever ask but allows SP to portray a fully developed patient personality. If none of the items below are particulars for the case, please write “all may be used.” | |
| Age range | 49 - 55 |
| Religious/spiritual background | all may be used |
| Sex (e.g., male, female, intersex, transwoman, transman) | female |
| Sexual orientation (e.g., heterosexual, lesbian, gay, bisexual, pansexual, queer, asexual) | all may be used |
| Gender expression (e.g., man, woman, genderqueer) | woman |
| Race and ethnicity (e.g., to promote educational diversity, we use a diverse pool of SPs.) | all may be used |
| Physical description (e.g., BMI, height range) | All may be used |
| Physical limitations | No |
| Patient appearance (e.g., disheveled, hospital gown, business casual, casual) | Tidy |
| Moulage + location (e.g., none, bruises, scars, body piercing, tattoos) | No |
| Affect (e.g., pleasant, cooperative) | A little bit frustrated and a little bit tired but pleasant and cooperative |
| Family group (e.g., who is family, who they live with) | Lives with husband, and daughters, 13 and 15  Married 18 years |
| Education | High school graduate and completed a 2-year associates degree. |
| Level of health literacy | Average |
| Employment, if any - present and past, noting any current stresses | Works as an executive assistant at a big accounting firm |
| Home/homeless - type of dwelling, number of stories, owned or rented | Single family home, owned |
| Financial situation - any current stresses | No |
| Insurance status (e.g., un/under/insured, public/private, HMO/PPO) | HMO insurance |
| Habits (i.e., diet, exercise, caffeine, smoking, alcohol, drugs) | Speed walks 3 miles/5 days a week, one cup of coffee daily, average American diet, nonsmoker, drinks wine at dinner 3 – 4 times a week, no illicit drug use. |
| Activities (i.e., hobbies, sports, clubs, friends) | Needlework |
| Typical day - what is the usual daily routine | Busy with work and keeping a home along with kids activities |

| **CASE INFORMATION** | |
| --- | --- |
| Chief Concern: What the patient will say when greeted by the student. The patient’s primary reason for seeking medical care often stated in their own words.  Opening Line:  Response to open-ended follow-up question:  Response to second open-ended follow-up question: | You are a 51 year/old executive assistant at a large accounting firm. You made an appointment with your doctor because you have been having hot flashes and difficulty sleeping.    Hi, is it hot in here? Can you turn the air conditioning on please?    I don’t know why I get so hot. Maybe this is menopause?  Oh my gosh. How long is this going to go on?! It stops, doesn’t it? Will I ever feel normal again? |
| **Additional Concerns**: Other, if any, concerns the patient has today (i.e., symptoms, requests, expectations, etc.) that will become part of set agenda. | You appear consumed by worry of a few things:   1. You have heard about “the change of life” and you fear this is what is happening. You have heard that everything for women goes downhill when this happens. 2. Some of your friends take hormones but you are worried because you hear that hormones cause cancer. 3. You are concerned your boss will notice that you are having hot flashes and ask you if you are OK. That would be so embarrassing. 4. You are afraid that your relationship with your husband will not be the same. |
| **THE PATIENT’S STORY**: The SP will be asked to tell their symptom story and the personal and emotion impact for each of their concerns. You will want to write this in the patient’s voice. The symptom story should be able to answer this question: “Tell me more about [chief concern/additional concern], starting at the beginning and bringing me up to now.”  The personal context should be able to answer questions concerning the broader personal/psychosocial context of symptoms, especially the patient’s beliefs/attributions.  The emotional context should be able to ask how are you doing with this, how does this make you feel, how has this affected you emotionally? IMPACT: How has this affected your life? How has this been for your family? | For the last 4 weeks you have been feeling hot and sweaty several times during the day and especially at night. You do not think you have a fever but this is really affecting your ability to get a good night’s sleep. You will wake up in the middle of the night and your pajamas are soaked. It seems to happen without warning and sometimes you feel some palpitations when it occurs. You went to a walk in clinic near where you work and you were told everything was fine except that your blood work showed signs of menopause. This wasn’t a great surprise to you because your periods have been spacing and you haven’t had one for 2 months.  You are also feeling fatigued, and you gained 5 pounds and don’t know why. You have not changed anything with your diet or exercise. You like to speed walk 3 miles five times a week. You have been cranky with your husband and daughters. You are used to doing 3 things at once and you are usually a very efficient person, but you have been feeling a bit forgetful. It is really bothering you that you can’t get as much done as you used to. You can no longer wear your warm sweater to work. You will be cold and then hot to the point that you want to tear off your clothes. |
| **HISTORY OF PRESENT ILLNESS**: Although some of the HPI will be given in the patient’s symptom story, the learners will expand the story during the direct question section. Below, describe the detailed history, usually about the chief concern, which the student must develop in order to make a useful assessment of the problem: | |
| Onset (when; gradual or sudden) | Gradual |
| Setting (what was going on or where was patient when symptoms first noticed?) | Difficulty sleeping and now also having symptoms during the day. |
| Duration (how long) | Began 4 weeks ago |
| Time relationships (frequency, constant or intermittent) | Because you can’t sleep well, you has been irritable and a little bit forgetful. You are not your usual efficient self at work, and you are cranky with your family. You feels more tired and hasn’t been able to exercise as much. |
| Location | -- |
| Radiation | -- |
| Quality | You are worried that you will not be the same and that your memory will fail, and you won’t be the same person with the same energy. |
| Amount | Hot flashes wake you up a few times at night. |
| Aggravated by what | Wearing sweaters and turtlenecks. |
| Relieved by what | Exercise helps a little. The symptoms seem to be better on the weekend |
| Attitude (what does the patient think is the problem, and how do they feel about it) | Worried that this won’t go away, and I am getting old. My husband is supportive and he listens but doesn’t know how to help me. He encouraged me to make this appointment today. |
| Overall course | You know this is probably the change of life and you don’t know when the symptoms will end. |
| REVIEW OF SYSTEMS: Significant positives and negatives | |
|  |  |
|  |  |
| **Past medical history** |  |
| Allergies to medications | None |
| Allergies to environmental/diet | None |
| Vaccinations | Uptodate on all recommended vaccinations |
| Medical Conditions | Hypothyroidism; mild anxiety - no meds, no prior treatment, or therapy |
| Pap | Yes, it was done this year and was normal. |
| Last menstrual period | My last period was 2 months ago. It was heavy and lasted a week. They usually last 5 days. |
| Last gynecological exam | 10 months ago and everything was OK |
| Surgeries | Your appendix was removed at age 19 |
| Accidents/injuries/trauma/illnesses | No |
| Last mammogram | Normal mammogram 6 months ago |
|  | |
| Inclusive sexual and reproductive history | |
| Sexual practices  Sexual partners  Protection: Use of safer sex practices  Use of birth control if appropriate  Risk of intimate partner violence | Sex is not as comfortable, so we haven’t been having it.  Husband is only partner  Feels safe in relationship |
| OB/GYN history | Age of onset of menses - 11  Age of menopause - now  Number of pregnancies - 2  Number of live births - 2  Number of miscarriages - 0  Number of abortions - 0 |
| Medications | Prescription/dose/reason - Synthroid 50 micrograms daily  Over the counter/dose/reason - none  Herbs/supplements/dose/reason - none  Other: |
| Immunizations – not included but she is uptodate | - Tetanus - Flu - Hepatitis - Pneumovax - HPV - Other |
| Tobacco products:   - Cigarettes - Cigar - Pipe - Chew - E-cigarettes | X Never   - Past - year started/year quit - Current   - Quantity   - # of years |
| Alcohol   - Beer   X Wine   - Liquor - Other | - Never - Past - year started/year quit   X Current   - Quantity one glass wine 3 – 4 times a week - # of years - 10 |
| Drugs   - Weed - Cocaine - Heroin - Meth - IV - Inhalants - Other | X Never   - Past - year started/year quit - Current   - Quantity   - # of years |
| Diet (describe) | Average American diet |
| Exercise (describe) | Speed walks 3 miles/5 times a week |
| List any other important social history or information important to this case | I tend to be a worrier. |
| Family history |  |
| Mother, father, siblings, grandparents, and other significant findings | Mother had early stage breast cancer at age 78 and is doing well now at 80. She tested negative for the BRCA gene. No family history of clots, ovarian cancer, osteoporosis.  Father deceased, 65, heart attack  1 brother, age 58, healthy |
|  |  |
| **Physical Exam** - List exam maneuvers expected for this case and any abnormal findings that SP will simulate. (tenderness, hyper-hypo reflex, rebound, weakness, etc.)  No physical exam performed | |
| PHYSICAL EXAM FINDINGS |  |
| 1. Written in layperson’s terms | No distress. |
| 2. General appearance - affect, appearance, position of patient at opening (i.e., sitting, lying down, holding abdomen, etc.) | Sitting in chair, fanning herself with a magazine. Cordial but having a hard time trying to cope with her symptoms while working and taking care of her family. Feeling a bit discouraged. |
| 1. Vital signs | HR 80, BP 122/78, T 98.6, RR 20, Pulse ox 98% |
| 2. Specific findings and affect | A little bit frustrated and a little bit tired. |
| 3. Response to certain physical movements | N/A |
|  |  |
| **DIAGNOSIS AND DIFFERENTIAL** |  |
| Diagnosis with support from positive and negative history and PE findings | Menopause transition with hot flashes and dropping estrogen levels. |
| Differential with support from positive and negative history and PE findings | Hypothyroidism, Anxiety |
|  |  |
| **MANAGEMENT OR DIAGNOSTIC PLAN** | Discussion of options to help her symptoms – conservative measures, nonhormonal medications, hormone replacement therapy. |
|  |  |
| **PROFESSIONALISM ISSUES OR CHALLENGES** | Conveying reassuring medical information and answering questions about misinformation that is in the public realm. |
|  |  |

| **If the learner asks…** | **Then, you answer** |
| --- | --- |
| What is your understanding of why you are here today/ the goal of today’s appointment? | I am having hot flashes and can’t sleep, and it is affecting my work and family. |
| How are you doing? | Worried that this won’t go away, and I am getting old. |
| Do you have any allergies? | No |
| Do you have any history of heart or lung problems? | No |
| Do you have any history of breast cancer, blood clots, or stroke? | No, but my mother was diagnosed with breast cancer at age 78 |
| Does anything make the hot flashes better? | Exercise helps a little. The symptoms seem to be better on the weekend |
| Does anything make the hot flashes worse? | Wearing sweaters and turtlenecks. |
| When were your thyroid hormones last checked? | The doctor at the clinic last week checked them and said they were fine. |
| Are you experiencing vaginal dryness? | Yes. Sex is not as comfortable, so we haven’t been having it. |
| Last gynecological exam? | 10 months ago |
| Do you have a history of mental/psychiatric disorders? | No, but I tend to be a worrier. |
| Is your pap screening up to date? | Yes, it was done this year and normal. |
| Are you having any vaginal bleeding? | My last period was 2 months ago. It was heavy and lasted a week. They usually last 5 days. |
| Have you talked with your husband about this? | Yes, and he listens but doesn’t know how to help me. He encouraged me to make this appointment today. |

**Case 2: *MedEdPORTAL* Standardized Patient Case Development Tool**

**Date**: 2/20/2023

**Primary Case Author**: Carolyn Davis, MD

**Secondary Case Author**: Madison Collazo, MD

**Standardized Patient Educator**: Meghan Willson, BS, CHSE

**Name of Case:** SP Script Case 2 – Abnormal bone density counseling

**Name of Educational and/or Assessment Activity**: *The Effectiveness of a Simulation-Based Menopause Education Program*

**Patient Name**: Felicia Watson

**Chief Complaint:** Discussion of abnormal DEXA results

Most Likely Diagnosis and Differential With Rationale From History and/or Physical Exam: Osteopenia

**Challenge Question**: At 5 min. announcement, if you haven’t brought up this question already, you should find a way to ask, “Aren’t there natural hormones I can take for my bones?” My friend takes natural bioidentical hormones from her doctor and pharmacist.

**Domains:** Check all that apply

X	Professionalism

X	Communication and Interpersonal Skills

X	Medical History

- Physical Exam

X	Shared Decision-Making

X	Patient Education

X	Clinical Reasoning

- Documentation
- Handoff
- Presentation
- Other:

**Type and Level of Learner**: OBGYN, Internal Medicine, and Family Medicine residents

**Case Objectives**: Please list specific objectives for each of the domains you have checked above:

1. Identify the most common symptoms of menopause.
2. Demonstrate competency using a performance checklist regarding evidence-based national guidelines for hormonal and nonhormonal treatment of menopause symptoms.
3. Demonstrate knowledge of appropriate treatment for low bone density during a simulated patient encounter and knowledge test.
4. Through role-playing, demonstrate shared decision-making about treatment of menopausal symptoms, hormone replacement, and management of the abnormal bone density study (DEXA).

| SETTING: outpatient, in patient, ED, home, nursing home, rehab, group, etc. | Consultation room |
| --- | --- |
| **PATIENT PROFILE:** Information about the “patient” that helps select an SP and helps the learner get an understanding of them as a person. SP will know more information about the patient than learner will ever ask but allows SP to portray a fully developed patient personality. If none of the items below are particulars for the case, please write “all may be used.” | |
| Age range | 58 - 64 |
| Religious/spiritual background | all may be used |
| Sex (e.g., male, female, intersex, transwoman, transman) | female |
| Sexual orientation (e.g., heterosexual, lesbian, gay, bisexual, pansexual, queer, asexual) | all may be used |
| Gender expression (e.g., man, woman, genderqueer) | woman |
| Race and ethnicity (e.g., to promote educational diversity, we use a diverse pool of SPs.) | all may be used |
| Physical description (e.g., BMI, height range) | BMI 18-25 |
| Physical limitations | No |
| Patient appearance (e.g., disheveled, hospital gown, business casual, casual) | Tidy, dressed in regular clothes |
| Moulage + location (e.g., none, bruises, scars, body piercing, tattoos) | No |
| Affect (e.g., pleasant, cooperative) | Pleasant, doesn’t like to take pills and she prefers to do things “naturally” |
| Family group (e.g., who is family, who they live with) | Married for 21 years, widowed. Now with current partner for 4 years. Two sons, ages 30 and 34 |
| Education | High school graduate |
| Level of health literacy | Average |
| Employment, if any - present and past, noting any current stresses | Works at Macy’s, womens clothing |
| Home/homeless - type of dwelling, number of stories, owned or rented | Single family home, owned |
| Financial situation - any current stresses | No |
| Insurance status (e.g., un/under/insured, public/private, HMO/PPO) | HMO insurance |
| Habits (i.e., diet, exercise, caffeine, smoking, alcohol, drugs) | Plays golf on weekends, doesn’t have much time to exercise during the week, one cup of coffee daily, average American diet, nonsmoker, Glass of wine 1 – 2 times/month, no illicit drug use. |
| Activities (i.e., hobbies, sports, clubs, friends) | Grows roses in her garden |
| Typical day - what is the usual daily routine | Busy with work, most days. Relaxes in her garden on weekends. |

| **CASE INFORMATION** | |
| --- | --- |
| **Chief Concern**: What the patient will say when greeted by the student. The patient’s primary reason for seeking medical care often stated in their own words.  Opening Line:  Response to open-ended follow-up question:  Response to second open-ended follow-up question: | You are sitting in the exam room in everyday clothes and waiting to talk to the doctor about your bone density test.    Hi, do you think I need to be worried about my bone density report?  I don’t want to break my hip like my father did. He was never able to walk after that happened.  My friend said she saw a news report about medications for osteoporosis and that they were dangerous. |
| Additional Concerns: Other, if any, concerns the patient has today (i.e., symptoms, requests, expectations, etc.) that will become part of set agenda. | You have a few questions and concerns:   1. You feel fine and don’t want to take a medication. You like natural treatments. 2. Some of your friends have told you the medications are dangerous. 3. Your dentist has told you to try to avoid the medications for osteoporosis because of problems with the jawbone. |
| **THE PATIENT’S STORY:** The SP will be asked to tell their symptom story and the personal and emotion impact for each of their concerns. You will want to write this in the patient’s voice. The symptom story should be able to answer this question: “Tell me more about [chief concern/additional concern], starting at the beginning and bringing me up to now.”  The personal context should be able to answer questions concerning the broader personal/psychosocial context of symptoms, especially the patient’s beliefs/attributions.  The emotional context should be able to ask how are you doing with this, how does this make you feel, how has this affected you emotionally? IMPACT: How has this affected your life? How has this been for your family? | You are a 60 year/old sales associate, working in the women’s department at Macys. You recently went to a health fair where they were offering a free bone density test. You were told you had osteopenia, so you made an appointment with your doctor because you don’t know what that means.  Your periods stopped 9 years ago and you have not had any bleeding since. You still occasionally have hot flashes. “Is this normal?”, you wonder. You have not taken any hormones. Your job keeps you busy and on your feet during the week. Overall, you feel healthy except for the occasional aches and pains. You have heard that women your age need calcium and Vit D, so you put milk in your coffee every morning and you get Vit D when you golf outside every weekend. |
| **HISTORY OF PRESENT ILLNESS:** Although some of the HPI will be given in the patient’s symptom story, the learners will expand the story during the direct question section. Below, describe the detailed history, usually about the chief concern, which the student must develop in order to make a useful assessment of the problem: | |
| Onset (when; gradual or sudden) | I feel OK but this report says I might break my bones. |
| Setting (what was going on or where was patient when symptoms first noticed?) | Free bone density was being offered at a health fair and I had the test. |
| Duration (how long) | -- |
| Time relationships (frequency, constant or intermittent) | -- |
| Location | -- |
| Radiation | -- |
| Quality | -- |
| Amount | -- |
| Aggravated by what | I want to be able to keep playing golf with my girlfriends on the weekend so I am a little worried. Should I not exercise? Don’t you have pain before you break a bone? |
| Relieved by what | -- |
| Attitude (what does the patient think is the problem, and how do they feel about it) | I really like being independent and taking care of myself. |
| Overall course | What will I do if I break a bone? If I take a medication, do I have to take it for life? |
| REVIEW OF SYSTEMS: Significant positives and negatives | |
|  |  |
|  |  |
| **Past medical history** |  |
| Allergies to medications | None |
| Allergies to environmental/diet | None |
| Vaccinations | Uptodate on all recommended vaccinations |
| Medical Conditions | Mild Hypertension  Reflux (GERD), mild |
| Pap | Yes, it was done this year and was normal. |
| Last menstrual period | My last period was 8 yr ago. No bleeding since. |
| Last primary care appointment | 10 days ago, everything was OK |
| Surgeries | None |
| Accidents/injuries/trauma/illnesses | No |
| Last mammogram | Normal mammogram 6 months ago |
| Inclusive sexual and reproductive history | |
| Sexual practices  Sexual partners  Protection: Use of safer sex practices  Use of birth control if appropriate  Risk of intimate partner violence | Infrequent sexual activity due to vaginal dryness.  One partner of 4 years.  Feels safe in relationship |
| OB/GYN history | Age of onset of menses - 11  Age of menopause - now  Number of pregnancies - 2  Number of live births - 2  Number of miscarriages - 0  Number of abortions - 0 |
| Medications | Prescription/dose/reason - HCTZ 25 mg daily  Over the counter/dose/reason - none  Herbs/supplements/dose/reason - none  Other: |
| Immunizations – not included but she is uptodate | - Tetanus - Flu - Hepatitis - Pneumovax - HPV - Other |
| Tobacco products:   - Cigarettes - Cigar - Pipe - Chew - E-cigarettes | X Never   - Past - year started/year quit - Current   - Quantity   - # of years |
| Alcohol   - Beer   X Wine   - Liquor - Other | - Never - Past - year started/year quit   X Current   - Quantity one glass wine 1 – 2 times/month - # of years - 10 |
| Drugs   - Weed - Cocaine - Heroin - Meth - IV - Inhalants - Other | X Never   - Past - year started/year quit - Current   - Quantity   - # of years |
| Diet (describe) | Average American diet |
| Exercise (describe) | Plays golf on weekends, doesn’t have much time to exercise during the week. |
| List any other important social history or information important to this case | I tend to be a worrier. |
| **Family history** |  |
| Mother, father, siblings, grandparents, and other significant findings | Mother had early stage breast cancer age 64, now well at 82  Father hip fracture from a fall on the ice at age 78, dec. at 80  2 sisters, 54 and 58, healthy  No family history of ovarian cancer or blood clots. |
|  |  |
| Physical Exam - List exam maneuvers expected for this case and any abnormal findings that SP will simulate. (tenderness, hyper-hypo reflex, rebound, weakness, etc.)  No physical exam performed | |
| **PHYSICAL EXAM FINDINGS** |  |
| 1. Written in layperson’s terms | No distress. |
| 1. General appearance - affect, appearance, position of patient at opening (i.e., sitting, lying down, holding abdomen, etc.) | Sitting in in the consultation room waiting to discuss results. |
| 2. Vital signs | HR 80, BP 135/85, BMI 19 |
| 1. Specific findings and affect | Normal affect |
| 2. Response to certain physical movements | N/A |
|  |  |
| **DIAGNOSIS AND DIFFERENTIAL** |  |
| Diagnosis with support from positive and negative history and PE findings | Osteopenia on DEXA with risk factors for fracture |
| Differential with support from positive and negative history and PE findings |  |
|  |  |
| **MANAGEMENT OR DIAGNOSTIC PLAN** | Discussion of options to help her symptoms – diet, exercise, calcium/Vit D recommendations, nonhormonal medications, hormonal medications as well as discussion of risks of treating versus not treating. |
|  |  |
| **PROFESSIONALISM ISSUES OR CHALLENGES** | Conveying reassuring medical information and answering questions about misinformation that is in the public realm. |
|  |  |

| **If the learners asks…** | **Then, you answer** |
| --- | --- |
| What is your understanding of why you are here today/ the goal of today’s appointment? | My bone density report says my bones might be weak. |
| How are you doing? | Worried because my father broke his hip and he did not do well after the surgery. |
| Do you have any allergies? | No |
| Do you have any history of heart, lung, or kidney problems? | No |
| Do you have any history of breast cancer, blood clots, or stroke? | No, but my mother was diagnosed with breast cancer at age 64 |
| How much calcium to you take daily? | I put milk in my coffee every morning. |
| Do you take any Vitamin D? | No…. do I have to? I heard you can get that from the sun. |
| Do you have any thyroid problems? | No |
| Have you ever been diagnosed with a stomach ulcer or reflux(heartburn) | No. I can’t eat close to bedtime. If I do, I have heartburn and must take a Gaviscon. |
| Last gynecological exam? | 10 months ago, and everything was normal. |
| Do you have a family history of hip fractures or osteoporosis? | My father broke his hip at age 78. |
| When was your last mammogram? | 7 months ago, and normal |
| Are you sexually active? | Not really. It is too dry. |
| Have you tried anything for the dryness? | KY jelly which helps a little |
| Are you having any problems with sleeping? | I don’t sleep as well as I used to. My partner likes to keep the TV on in the bedroom. |

**Case 3: *MedEdPORTAL* Standardized Patient Case Development Tool**

**Date**: 2/20/2023

**Primary Case Author:** Carolyn Davis, MD

**Secondary Case Author**: Madison Collazo, MD

**Standardized Patient Educator**: Meghan Willson, BS, CHSE

**Name of Case:** SP Script Case 3 - GSM Counseling

**Name of Educational and/or Assessment Activity:** *The Effectiveness of a Simulation-Based Menopause Education Program*

**Patient Name:** Doretha Patel

**Chief Complaint:** Vaginal dryness and painful sex

**Most Likely Diagnosis and Differential With Rationale From History and/or Physical Exam**: Genitourinary Syndrome of Menopause

**Challenge Question:** At 5 min. announcement, if you haven’t brought up this question already, you should find a way to ask “What about the “safe natural bioidentical hormones”? My friend tells me that her pharmacist makes hormone cream for her. (Please say both sentences so that the resident learner knows that you are referring to compounded bioidentical hormones.)

**Domains: Check all that apply**

X	Professionalism

X	Communication and Interpersonal Skills

X	Medical History

□	Physical Exam

X	Shared Decision-Making

X	Patient Education

X	Clinical Reasoning

□	Documentation

□	Handoff

□	Presentation

□	Other:

**Type and Level of Learner**: OBGYN, Internal Medicine, and Family Medicine residents

**Case Objectives**: Please list specific objectives for each of the domains you have checked above:

1. Identify the most common symptoms of menopause.
2. Demonstrate competency using a performance checklist regarding evidence-based national guidelines for hormonal and nonhormonal treatment of menopause symptoms.
3. Demonstrate knowledge of appropriate treatment for low bone density during a simulated patient encounter and knowledge test.
4. Through role-playing, demonstrate shared decision-making about treatment of menopausal symptoms, hormone replacement, and management of the abnormal bone density study (DEXA).

| **SETTING**: outpatient, in patient, ED, home, nursing home, rehab, group, etc. | Outpatient exam room |
| --- | --- |
| **PATIENT PROFILE**: Information about the “patient” that helps select an SP and helps the learner get an understanding of them as a person. SP will know more information about the patient than learner will ever ask but allows SP to portray a fully developed patient personality. If none of the items below are particulars for the case, please write “all may be used.” | |
| Age range | 55 - 60 |
| Religious/spiritual background | all may be used |
| Sex (e.g., male, female, intersex, transwoman, transman) | female |
| Sexual orientation (e.g., heterosexual, lesbian, gay, bisexual, pansexual, queer, asexual) | all may be used |
| Gender expression (e.g., man, woman, genderqueer) | woman |
| Race and ethnicity (e.g., to promote educational diversity, we use a diverse pool of SPs.) | all may be used |
| Physical description (e.g., BMI, height range) | All may be used |
| Physical limitations | No |
| Patient appearance (e.g., disheveled, hospital gown, business casual, casual) | Tidy dressed in regular work clothes sitting in a chair |
| Moulage + location (e.g., none, bruises, scars, body piercing, tattoos) | No |
| Affect (e.g., pleasant, cooperative) | Pleasant and slightly worried |
| Family group (e.g., who is family, who they live with) | Divorced. Lives alone  Two sons, ages 19 and 23 away at school |
| Education | Finished college |
| Level of health literacy | Average |
| Employment, if any - present and past, noting any current stresses | Teaches math in a local high school. |
| Home/homeless - type of dwelling, number of stories, owned or rented | Condo, owned |
| Financial situation - any current stresses | No |
| Insurance status (e.g., un/under/insured, public/private, HMO/PPO) | PPO insurance |
| Habits (i.e., diet, exercise, caffeine, smoking, alcohol, drugs) | Peloton bike workouts 3 times a week, one cup of coffee daily, mediterranean diet, nonsmoker, drinks wine 1 – 2 times a month, no illicit drug use. |
| Activities (i.e., hobbies, sports, clubs, friends) | Biking |
| Typical day - what is the usual daily routine | Teaches high school, relaxes on the weekends. |

| **CASE INFORMATION** | |
| --- | --- |
| **Chief Concern**: What the patient will say when greeted by the student. The patient’s primary reason for seeking medical care often stated in their own words.  Opening Line:  Response to open-ended follow-up question:  Response to second open-ended follow-up question: | You are a 55 year/old high school teacher. You made an appointment with your doctor because you have been experiencing pain with intercourse and therefore you have no interest in sex. You just had a gyn exam with your doctor. Now you are dressed in your clothes and sitting in the exam room waiting to discuss a treatment plan.  I am hoping you can help me. I have a new partner and sex is not comfortable.  There is a lot of vaginal dryness, and he is a couple of years younger than me. I am worried he might leave me because of this.  I am not sure what I can do, you just hear so much about hormones and cancer. |
| **Additional Concerns**: Other, if any, concerns the patient has today (i.e., symptoms, requests, expectations, etc.) that will become part of set agenda. | You appear consumed by worry of a few things:   1. You wonder if this is just how it’s going to be and that makes you kind of sad now that you have a new partner. You wonder if he will leave you. 2. Some of your friends take hormones but you are worried because you hear that hormones cause cancer. |
| **THE PATIENT’S STORY**: The SP will be asked to tell their symptom story and the personal and emotion impact for each of their concerns. You will want to write this in the patient’s voice. The symptom story should be able to answer this question: “Tell me more about [chief concern/additional concern], starting at the beginning and bringing me up to now.”  The personal context should be able to answer questions concerning the broader personal/psychosocial context of symptoms, especially the patient’s beliefs/attributions.  The emotional context should be able to ask how are you doing with this, how does this make you feel, how has this affected you emotionally? IMPACT: How has this affected your life? How has this been for your family? | You have been menopausal since the age of 52 and the hot flashes have mostly subsided. You are in a new relationship and recently became sexually active. However, sex has been uncomfortable, so you have not been that interested in having sex. Your significant other has been very understanding. Everything feels dry down there and KY jelly is not helping much. |
| **HISTORY OF PRESENT ILLNESS**: Although some of the HPI will be given in the patient’s symptom story, the learners will expand the story during the direct question section. Below, describe the detailed history, usually about the chief concern, which the student must develop in order to make a useful assessment of the problem: | |
| Onset (when; gradual or sudden) | Gradual |
| Setting (what was going on or where was patient when symptoms first noticed?) | You are in a new relationship and recently became sexually active. However, sex has been uncomfortable, so you have not been that interested in having sex. |
| Duration (how long) | A couple of years ago but noticing more now that I have a partner. |
| Time relationships (frequency, constant or intermittent) | Now that I have a partner, it would be nice to have nonpainful sex. The dryness is really uncomfortable, and it makes me not interested in having sex. |
| Location | -- |
| Radiation | -- |
| Quality | My partner listens but he doesn’t know how to help me. He encouraged me to make this appointment today. |
| Amount | -- |
| Aggravated by what | Summer’s Eve cleansing wipes really burn. |
| Relieved by what | Just using water in the area to clean. Maybe KY jelly helps a little but not enough. |
| Attitude (what does the patient think is the problem, and how do they feel about it) | What if I never can have sex again? Will my partner leave me? |
| Overall course | I would be really sad if I couldn’t have a normal physical relationship. It would be very lonely. |
| REVIEW OF SYSTEMS: Significant positives and negatives | |
|  |  |
|  |  |
| **Past medical history** |  |
| Allergies to medications | None |
| Allergies to environmental/diet | None |
| Vaccinations | Uptodate on all recommended vaccinations |
| Medical Conditions | Mild Asthma |
| Pap | I was told I didn’t need paps anymore |
| Last menstrual period | Age 42 after the hysterectomy |
| Last gynecological exam | 10 months ago and everything was OK |
| Surgeries | Laparoscopic hysterectomy at age 42 – fibroids  Ovaries left in |
| Accidents/injuries/trauma/illnesses | Kidney stone age 48 |
| Last mammogram | Normal mammogram 6 months ago |
| History of bladder infections one last year | |
| Inclusive sexual and reproductive history | |
| Sexual practices  Sexual partners  Protection: Use of safer sex practices  Use of birth control if appropriate  Risk of intimate partner violence | Has a new partner for 2 months.  Feels safe in relationship |
| **OB/GYN history** | Age of onset of menses - 11  Age of menopause - now  Number of pregnancies - 3  Number of live births - 2  Number of miscarriages - 1  Number of abortions - 0 |
| Medications | Prescription/dose/reason - Singular 10 mg daily  Over the counter/dose/reason - none  Herbs/supplements/dose/reason - none  Other: |
| Immunizations – not included but she is uptodate | - Tetanus - Flu - Hepatitis - Pneumovax - HPV - Other |
| Tobacco products:   - Cigarettes - Cigar - Pipe - Chew - E-cigarettes | X Never   - Past - year started/year quit - Current   - Quantity   - # of years |
| Alcohol   - Beer   X Wine   - Liquor - Other | - Never - Past - year started/year quit   X Current   - Quantity one glass wine 1 - 2 times a month - # of years - 20 |
| Drugs   - Weed - Cocaine - Heroin - Meth - IV - Inhalants - Other | X Never   - Past - year started/year quit - Current   - Quantity   - # of years |
| Diet (describe) | Mediterranean diet |
| Exercise (describe) | Pelaton bike workouts 3 times a week |
| List any other important social history or information important to this case | I tend to be a worrier. |
| **Family history** |  |
| Mother, father, siblings, grandparents, and other significant findings | Mother had early stage breast cancer at age 78 and is doing well now at 80. She tested negative for the BRCA gene. No family history of osteoporosis, ovarian cancer, hip fractures, or blood clots  Father - Alive, 80, Parkinson’s Disease  One brother, 60, alive and well |
|  |  |
| **Physical Exam** - List exam maneuvers expected for this case and any abnormal findings that SP will simulate. (tenderness, hyper-hypo reflex, rebound, weakness, etc.) – **physical exam not done but the findings below are on the door notes for the student/resident learner.**  Exam findings:  External genitalia – normal female anatomy  Vagina - pale and dry vulvovaginal mucosa with petechiae, loss of vaginal rugae, no discharge seen, absent cervix.  Bimanual exam – absent uterus, no adnexal masses. | |
| PHYSICAL EXAM FINDINGS |  |
| 1. Written in layperson’s terms | No distress. |
| 2. General appearance - affect, appearance, position of patient at opening (i.e., sitting, lying down, holding abdomen, etc.) | Sitting in chair, feeling a bit discouraged. |
| 1. Vital signs | HR 78, BP 122/75, T 98.6, RR 20 |
| 2. Specific findings and affect | Normal affect |
| 3. Response to certain physical movements | N/A |
|  |  |
| **DIAGNOSIS AND DIFFERENTIAL** |  |
| Diagnosis with support from positive and negative history and PE findings | Genitourinary Syndrome of Menopause |
| Differential with support from positive and negative history and PE findings |  |
|  |  |
| **MANAGEMENT OR DIAGNOSTIC PLAN** | Discussion of options to help her symptoms – conservative measures, nonhormonal medications, hormone therapy. |
|  |  |
| **PROFESSIONALISM ISSUES OR CHALLENGES** | Conveying reassuring medical information and answering questions about misinformation that is in the public realm. |
|  |  |

| **If student asks…** | **Then, you answer** |
| --- | --- |
| What is your understanding of why you are here today/ the goal of today’s appointment? | I am having pain with intercourse and it makes me not want to have sex. |
| How are you doing? | Worried that this won’t go away, and I am getting old. |
| Do you have any allergies? | No |
| Do you have any history of heart or lung problems? | No |
| Do you have any history of breast cancer, blood clots, or stroke? | No |
| Does anything make the symptoms better? | Just using water in the area to clean. Maybe KY jelly helps a little but not enough. |
| Does anything make the symptoms worse? | Summer’s Eve cleansing wipes really burn. |
| Do you have any history of medical problems? | I have asthma but it is mild. |
| Have you had any surgeries? | I had a hysterectomy at age 42 for fibroids. |
| Last annual routine gynecological exam? | 10 months ago |
| Is your pap screening up to date? | I was told I didn’t need paps anymore |
| When was your last mammogram? | 6 months ago, and normal |
| Have you talked with your partner about this? | Yes. He listens but he doesn’t know how to help me. He encouraged me to make this appointment today. |
| When was your last period? Have you had any bleeding since? | Hysterectomy at age 42. Last period was age 42. I kept my ovaries. |
| Do you have any bleeding with sex? | No |
| Are you having any problems with sleeping? | I don’t sleep as well as I used to. My partner likes to keep the TV on in the bedroom. |

*****If the resident spends a long time discussing your history and does not explain the options of nonhormonal and hormonal treatments, it is OK to prompt them by saying, “can we talk about the treatments and things I can do to feel better down there”

*****If a student does not offer alternatives to hormones, you should ask, “do I have to take hormones? Is there nothing else I can try?”
